# The Impact of Threat Level and Performance Feedback on Checking Behaviour and Its Concomitants: A Multi-Site Study

**DOI:** 10.1177/17470218261456728

**Published:** 2026-05-26

**Authors:** Sruthi Sridhar, Bronwyn Steenekamp, Anthea Manuel, Belkis Sayin, Nicolò Biagi, Shannon Wake, Jayne Morriss

**Affiliations:** 1 School of Psychology and Clinical Language Sciences, University of Reading, UK; 2 School of Psychology, Faculty of Environmental and Life Sciences, University of Southampton, UK; 3 Henley Business School, Business Informatics Systems and Accounting, Informatics Research Centre, University of Reading, UK

**Keywords:** threat, performance feedback, checking, corrugator supercilii

## Abstract

Checking behaviours are commonly engaged to prevent adverse outcomes and alleviate uncertainty. Understanding how community populations engage in checking behaviours based on different factors such as threat level and performance feedback may inform us as to how checking behaviours become excessive in clinical obsessive–compulsive disorder populations. To address these questions, this study manipulated threat level (low threat: performance score only; high threat: mild electric shock based on performance score) and performance feedback (no feedback, feedback) during a visual discrimination and checking task. During the task, the participants’ goal was to identify whether two shapes presented in succession were identical or different. Throughout the task, we recorded multiple read out measures: checking frequency, ratings of urge to check and distress, task performance, and corrugator supercilii activity. Data were recorded across two sites (*n* = 208). Higher versus lower-threat level, increased checking behaviour at one site but not the other. Furthermore, higher versus lower-threat levels improved task performance for identifying identical shapes versus different shapes. In addition, no feedback, compared to feedback, was associated with greater corrugator supercilii activity and improved task performance when identifying identical shapes versus different shapes. Overall, these findings provide insight into how factors such as threat level and performance feedback differently impact checking behaviour and its concomitants in community samples.

## Introduction

Safety-seeking behaviours such as escape and avoidance are enacted to prevent or minimise the effects of threat (Tashjian et al., 2022; van Uijen & Toffolo, 2015; Wake et al., 2022; Wong et al., 2023). A common type of safety-seeking behaviour is checking behaviour, which involves the repetition of an action which was thought to be performed incorrectly or failed to be performed entirely. Individuals may engage in checking behaviour to gather further information in order to alleviate uncertainty or negative consequences (Biria et al., 2024; Bottesi et al., 2017; Cuttler & Graf, 2008; Rotge et al., 2015; Wong et al., 2023). While checking behaviours can have adaptive purposes, unnecessary and compulsive checking are central to obsessive–compulsive disorders (OCDs; Rachman, 2002). In Rachman’s (2002) seminal model, checking behaviour is thought to be underpinned by heightened responsibility for preventing harm, doubt, and lower confidence in memory. Prior research has used a variety of methods to capture checking behaviours, including semi-structured interviews (Parrish & Radomsky, 2010), vignette-based tasks (Parrish & Radomsky, 2011), goal-oriented tasks with potential threat (e.g. operating kitchen apparatus; Bucarelli & Purdon, 2016), and visual discrimination tasks (Morein-Zamir et al., 2018; Wake et al., 2022). These studies indicate that checking behaviours are present in both community and non-clinical samples (Parrish & Radomsky, 2011; Wake et al., 2022), suggesting that checking is a common behavioural response. However, individuals with subclinical (Toffolo et al., 2013) or clinical OCD (Clair et al., 2013; Rotge et al., 2008) display more frequent and persistent checking. The level of situational threat based on specific environmental conditions (e.g. Naylor et al., 2015; Pepperdine et al., 2018) may influence the extent of checking behaviour (Strauss et al., 2020). Research has demonstrated that when threatened with punishment, or the loss of a reward, individuals engage in more frequent, prolonged checking (Li et al., 2016; Morein-Zamir et al., 2018). Furthermore, greater perceived threat results in a higher self-reported urge to check (Parrish & Radomsky, 2011). However, past research has not directly examined how the level of threat parametrically (e.g. lower-threatening outcome versus a higher-threatening outcome such as a mild electric shock) impacts checking frequency. Thus, it is difficult to ascertain whether higher levels of actual threat lead to greater checking behaviour in a linear way.

Uncertainty can be considered a type of threat (Hewitt et al., 2009). Importantly, uncertainty can be reduced through gathering information from the environment passively via external feedback or actively by engaging in behaviours such as checking (Bottesi et al., 2017; Brydevall et al., 2018; Rotge et al., 2015; Toffolo et al., 2013). Prior research has established that under high uncertainty, there is greater desire for performance feedback (Anseel & Lievens, 2007). Moreover, previous findings suggest that individuals differ in how to they react to negative or positive performance feedback and how this influences checking (Anseel & Lievens, 2007; Parrish & Radomsky, 2011). However, there is a lack of research examining how the presence or absence of performance feedback influences checking behaviour. In particular, little is known about the effects of performance feedback both on its own and within the context of threat. Therefore, it is difficult to infer whether the absence of performance feedback and threat are similarly dominant in driving checking behaviour, or whether the interaction of these two dimensions results in more significant checking behaviours.

The current study intends to address several gaps in the checking literature. Firstly, the study directly examines how different levels of threat and performance feedback, and their interaction, may contribute to checking behaviour, with the aim of clarifying the conditions under which checking might become elevated. Secondly, the study investigates how threat, performance feedback, and their interaction, map onto multiple readout measures, including checking behaviour, self-reported distress and urge to check, task performance (reaction time and accuracy), and corrugator supercilii activity. Multimodal examinations offer a more holistic view of checking behaviour phenomena. For instance, as far as we are aware, only one previous study has examined the corrugator supercilii in relation to checking behaviour (Wake et al., 2022). However, the corrugator supercilii may be an interesting measure to examine in relation to threat and performance feedback, as it is sensitive to stimuli that evoke distress and/or effort (Cacioppo et al., 1985, 1986). Thirdly, the study explores these effects within a community sample, which may facilitate a broader understanding of how checking behaviour operates outside clinical populations (Strauss et al., 2020) and may enhance the generalisability of the findings.

Extending previous research, in this study we modified a visual discrimination and checking based task (Wake et al., 2022) to examine the effects of different levels of threat and performance feedback. The level of threat was manipulated by allocating participants to separate experimental groups (between-participants) that had different types of consequences based on task performance (low threat: the task performance score was provided with no additional outcome; high threat: a lower task performance score could result in receiving an electric shock). The operationalisation of the low- and high-threat experimental groups was based on past research that suggests that both task performance monitoring (Elliot & Harackiewicz, 1996) and the anticipation of future electric shocks (Averill & Rosenn, 1972; Bublatzky et al., 2017) can independently evoke threat responses. Thus, the low-threat experimental group would experience potential threat from performance monitoring only, while the high-threat experimental group would experience potential threat from both performance monitoring and anticipating a future electric shock. The level of performance feedback was manipulated (within-participants) by altering the type of feedback participants received throughout the task (no feedback, feedback), in order to introduce uncertainty (e.g. no feedback) and certainty (e.g. feedback) about task performance. As in previous studies using similar task designs (Biria et al., 2024; Clair et al., 2013; Rotge et al., 2008; Wake et al., 2022), there were two shape types presented (identical, different). Throughout the task, crucial information as to how levels of threat and performance feedback impact subjective (i.e. ratings of the urge to check, ratings distress), behavioural (i.e. checking frequency, task accuracy, task reaction time), and physiological (i.e. corrugator supercilii activity) components related to checking was collected by recording multimodal responses.

We hypothesised that:

1. Given that checking is a commonly used as a safety-seeking behaviour to reduce threat (Cuttler & Graf, 2008), we predicted that individuals placed into the high-threat condition, compared to the low-threat condition, would exhibit increased checking frequency, ratings of the urge to check, and ratings of distress. Furthermore, we predicted that individuals would display higher task accuracy (for discussion see, Torrisi et al., 2016), slower task reaction time when giving a final answer, and greater corrugator supercilii activity (e.g. related to distress or effort, see Cacioppo et al., 1986).
2. For feedback, we predicted that that in the no feedback condition compared to the feedback condition, participants would show greater checking frequency, ratings of the urge to check, ratings of distress, corrugator supercilii (e.g. related to distress or effort, see Cacioppo et al., 1985), and poorer task accuracy (see Moret-Tatay et al., 2016; Morriss et al., 2022b). However, due to limited prior research, we did not make specific directional hypotheses for task reaction time when providing a final answer to each trial.
3. We predicted an interaction between threat level and performance feedback on the outcome measures. However, *r*, we did not make specific directional predictions for this interaction due to the sparsity of research on both these factors.
4. Based on prior literature (Rotge et al., 2008), we predicted that in the identical shape condition compared to different shape condition, participants would show greater checking frequency. Additionally, as reported in Wake et al. (2022), we predicted that in the identical shape condition compared to different shape condition, participants would display higher accuracy. These two predictions served as manipulation checks to assess whether the task was operating as expected. We further predicted that shape type may impact the other outcome measures. However, we did not make specific directional predictions due the lack of research reporting this factor in relation to subjective and physiological measures

## Methods

Data were collected across two sites in the United Kingdom (Reading and Southampton). The methods across the two sites were identical, except for the equipment used for the electric stimulation build-up procedure and the corrugator supercilii recording. These differences are stated below under the relevant sections.

### Participants

Two hundred and eight participants took part (Reading: 106; Southampton: 102; see Table 1). All participants were free of psychotropic medication, between the ages of 18 and 40 years old, and had no previous history of traumatic brain injury. Participants were recruited through flyers posted around the University of Reading and Southampton campuses, the university’s research participation pool (Sona), and word of mouth. Participants were rewarded 24 credits or £20 for 2 hrs of their time. Ethical approval was granted by the respective University Ethics Committees for the University of Reading (UREC 23/16) and the University of Southampton (78272).

**Table 1.** Demographics and OCI-R Scores Based on Group Allocation by Site.

	Reading		Southampton	
Variable	Low threat	High threat	Low threat	High threat
Age, mean (*SD*)	24 (4)	23 (2)	22 (4)	23 (4)
Sex (*n*)				
Female	30	26	38	33
Male	24	26	14	16
Sexual diversity (*n*)				
Heterosexual	40	27	29	39
LGBTQ	8	9	13	9
Not specified	6	16	10	1
Ethnicity (*n*)				
White	22	16	25	25
Asian	21	29	12	17
Mixed	2	1	3	3
Black	2	1	2	2
Not specified	7	5	10	2
Nationality (*n*)				
European	26	22	27	28
Asian	19	25	19	18
African	4	2	1	0
Middle Eastern	3	0	3	1
North American	1	0	1	1
Caribbean Islands	0	0	1	1
Not specified	1	3	0	0
OCI-R, mean (*SD*)	25.22 (12.34)	25.58 (12.53)	22.94 (12.30)	22.24 (11.66)

*Note. SD*: standard deviation; OCI-R: obsessive–compulsive inventory-revised.

The hypotheses were not preregistered. The experimental data were analysed using multilevel models (MLMs). MLMs are more powerful than ANOVAs as they can handle missing data (Quené & Van den Bergh, 2004). However, due to the complexity of MLMs, there is no agreed upon standard way of calculating power (Peugh, 2010). For this reason, the sample size estimate for each site was based on a power analysis for an ANOVA (repeated measures, within-between interaction).

The power analysis was conducted using G*Power 3.1.9.7 (Faul et al., 2007). The following parameters were used: effect size *f* = 0.15, alpha error probability = .05, Power (1 − β error probability) = .8, number of groups = 2 (low threat, high threat), number of measurements = 4 (2 [feedback, no feedback] × 2 (identical, different)), and correlation among repeated measures = .2. A small effect size was entered due to the understudied nature of this research area. The total sample size required per site was *n* = 100.

### Materials

#### Questionnaires

##### Obsessive–Compulsive Inventory

To assess obsessive and compulsive symptoms, we administered the Obsessive–Compulsive Inventory-Revised (OCI-R; Foa et al., 2002). The OCI-R consists of 18 items rated on a 5-point Likert Scale. All subscales (Checking, Hoarding, Neutralising, Obsessing, Ordering, and Washing) were included in the total score. A cutoff score of 21 is used to determine clinical levels of OCD. For means and standard deviations for each site, see Table 1. The internal consistency of OCI-R for each site: Reading: α = .878; Southampton: α = .889.

The balancing of OCI-R across low- and high-threat groups was performed using the clinical cutoff scores to reduce the confounding effects of individual differences in OCD symptoms, including those related to checking behaviour such as neutralising and obsessing. The total OCI-R score (site: *t*[206] = −1.66, *p* = .098, *d* = −0.23; threat: *t*[205.86] = 0.10, *p* = .924, *d* = 0.01) and OCI-R Checking subscale (site: *t*[205.93] = −0.30, *p* = .761, *d* = −0.04; threat: *t*[205.92] = −0.47, *p* = .639, *d* = −0.07) did not differ between groups or site.

### Visual Discrimination and Checking Task

The visual discrimination and checking task (see Figure 1A) was based on a previous checking task by our research group (Wake et al., 2022). The task was built using E-Prime 3.0 software (Psychology Software Tools Ltd., Pittsburgh, PA, United States). Visual stimuli were presented using a screen resolution of 800 × 600 with a 60 Hz refresh rate. Participants sat approximately 60 cm from the computer screen. Visual stimuli consisted of three different geometric shapes: a circle with an x in its centre, a cross, and a three-pointed star. All the shapes presented as visual stimuli throughout the task had a height and width of 50 mm.

**Figure 1.**
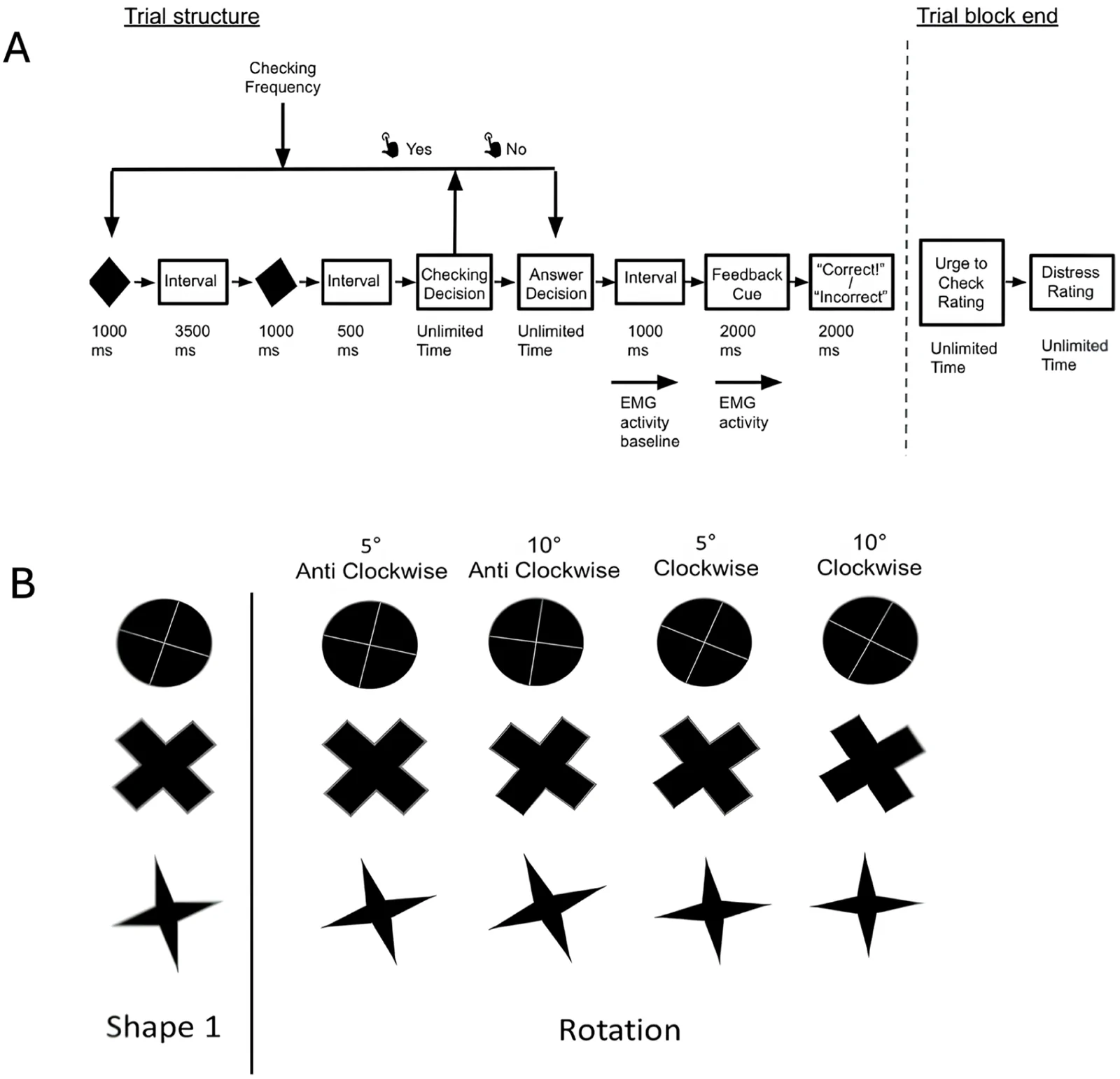
Trial structure and trial block end of the visual discrimination and checking task (A). In the trial structure, checking frequency was measured at the checking decision period, task accuracy and reaction time was measured at the answer decision period, and EMG – corrugator supercilii activity was recorded at the feedback cue period (displayed that no feedback was available or that feedback was coming). At the end of the trial block end, the urge to check and distress ratings were collected. Intervals represent blank screens. Shape stimuli used during the visual discrimination and checking task (B). Image reproduced from Wake et al. (2022). *Note.* EMG: electromyography; ms: milliseconds.

All trials began with the presentation of one of the three shapes on the computer screen for 1,000 ms. The shape then disappeared, and a white fixation cross in the centre of the screen was presented for an interval of 3,500 ms, after which a second shape was presented for 1,000 ms. The second shape was either identical to or different from the first shape. The different shapes consisted of one of four slightly rotated variations of the first shape: 5 degrees clockwise, 10 degrees clockwise, 5 degrees anticlockwise, and 10 degrees anticlockwise (see Figure 1B). The number of trials with identical stimuli was equal to the number of trials with different stimuli.

After a 500-ms interval following the presentation of the second shape, participants were given the opportunity to check whether the visual stimuli were identical or different, or they could choose to proceed to the choice phase. If the participant chose to check, the trial was repeated from the presentation of the first shape until they were presented with the option to check again. There was no restriction on the number of checks and no time limit on providing a response during either the check or choice phase. After the participant decided whether the shapes were identical or different, a short blank black screen was presented for 1,000 ms. Then a feedback instruction was presented for 2,000 ms, which either said, “No feedback available” or “Feedback is coming.” When feedback was available, a further screen displayed “Correct!” or “Incorrect” for 2,000 ms. At the end of each trial, a blank black screen was presented for 3,500 to 5,000 ms.

The task comprised 48 trials, with 12 trials per within-participants condition (identical stimuli with feedback, identical stimuli without feedback, different stimuli with feedback, different stimuli without feedback; see Table 2). There were 2 blocks: 24 trials with feedback and 24 trials without feedback. The blocks of trials were counterbalanced across participants, and the trials within each block were presented in a random order. The presentation of the shapes used as visual stimuli was equally distributed across the four conditions. For instance, for the identical stimuli with feedback and the identical stimuli without feedback, 12 identical shape pairs were presented (i.e. 4 circles, 4 crosses, and 4 stars).

**Table 2.** Summary of Experimental Conditions by Threat Level, Feedback, and Shape Type.

Condition	With feedback	Without feedback
Low threat (performance score presented at the end of the task)	Identical shape: 12 trials with performance feedback available Different shape: 12 trials with performance feedback available	Identical shape: 12 trials without performance feedback available Different shape: 12 trials without performance feedback available
High threat (potential for electric stimulation based on performance score at the end of the task)	Identical shape: 12 trials with performance feedback available Different shape: 12 trials with performance feedback available	Identical shape: 12 trials without performance feedback available Different shape: 12 trials without performance feedback available

*Note.* Low-threat and high-threat conditions were between-participants. Feedback and shape type were within-participants. There were 12 trials in each within-participants condition.

At the end of each block of trials, participants were asked to provide subjective ratings of “To what extent do you feel the urge to go back before making your decision?,” on a scale that ranged from 0 (“not at all”) to 100 (“extremely”). Participants were also asked to provide ratings of “How distressed did you feel during the last block of trials?” on a scale that ranged from 0 (“not at all”) to 100 (“extremely”).

At the end of the task, the computer displayed “You got most of the trials correct” to the low-threat condition and “Because you got most of the trials correct you will not receive a stimulation” to the high-threat condition.

### Electric Stimulation Equipment

At the Reading site, stimulation was delivered via a PowerLab 26T Isolated Stimulator (AD Instruments, Oxford, United Kingdom) using a MLADDF30 (AD Instruments) stimulating bar electrode that was attached to the little finger of the left hand.

At the Southampton site, stimulation was delivered using a STM200 (Biopac Systems, Norfolk, United Kingdom) constant voltage stimulator with a unipolar pulse. The stimulator was shielded from magnetic interference and grounded through an RF filter. Two touchproof LEAD100A snap leads were connected to EL502 stimulation sensors (Biopac Systems), which were attached to the participant’s left wrist for stimulation.

### Corrugator Supercilii Equipment

Measurements of the left corrugator supercilii were obtained by using two pairs of 4 mm Ag/AgCl bipolar surface electrodes connected to the ML138 Bio Amp (AD Instruments) at the Reading site or the MP160 system, and EMG100C add-on (Biopac Systems) at the Southampton site. The bipolar surface electrodes were approximately 15-mm apart. The ground electrode was a singular 8 mm Ag/AgCl electrode, placed upon the middle of the forehead, and connected to the ML138 Bio Amp (AD Instruments) at the Reading site or the EMG100C add-on (Biopac Systems) at the Southampton site. Corrugator supercilii activity was sampled at 1,000 Hz. A high-pass filter of 20 Hz was applied to the raw corrugator supercilii signal online (Solnik et al., 2008).

### Procedure

Upon arrival at the laboratory, participants were informed about the experimental procedure and asked to complete a consent form. They were seated in the testing booth, where they completed questionnaires on a computer. Next, participants were asked to wash their hands without soap and return to the testing booth. Skin sites for the corrugator supercilii and ground electrodes were then lightly abraded with isopropyl alcohol skin prep pads to reduce skin impedance, after which electromyography sensors were attached to the left corrugator supercilii. Skin-conductance sensors (not reported here) were attached to the participants’ index and middle fingers of the left hand.

To ensure that an even number of participants with low and high checking tendencies were allocated to each experimental group (low threat; high threat), a research assistant assigned each participant to one of the relevant experimental groups before each task. Low and high checking tendencies were defined as scores below or above the average for the checking items of the OCI-R (Foa et al., 2002).

Participants allocated to the low-threat condition proceeded directly to the visual discrimination and checking task. Participants allocated to the high-threat condition first completed the electric stimulation build-up procedure The researcher explained the procedure verbally and informed the participants that “the intensity of the stimulation would be set by you,” and they were instructed to determine a level that was “highly unpleasant, but not painful.” Participants received an initial mild stimulation (Reading: repeat rate of 20 Hz, repeats 10, pulse width of 0.2 ms, starting at 1 mA; Southampton: 500 ms pulse width, starting at 5 V), which was gradually increased according to their self-reported intensity and pain. The research assistant confirmed willingness to increase the level before each stimulation was delivered. The participant was then prompted by the researcher to rate the intensity and pain of the stimulation on a scale from 0 to 10 verbally, where 0 was “not painful at all” and 10 was “extremely painful” (Reading: *M* = 6.37; *SD* = 1.92; Southampton: *M* = 6.57, *SD* = 1.28, four participants at Southampton did not verbally report a pain rating). Once the participant reached a suitable level, the stimulation was set for the duration of the experiment (Reading: *M* = 6.67 mA; *SD* = 3.14 mA; Southampton: *M* = 28.47 V; *SD* = 8.25 V). The stimulation electrodes remained attached to the participant until the end of the experiment to preserve the credibility of the threat. At the start of the visual discrimination and checking task, participants in the high-threat condition were informed: “Depending on your task performance (e.g. how many trials you get correct), you may or may not receive a mild electric stimulation at the end of the task.” Participants in the low-threat condition were given no such instructions. Participants were instructed to remain as still as possible, maintain attention, and respond using the keys on the keyboard. The task began with two practice trials (one identical, one different) using square-shaped stimuli, following the same trial structure described in the “Materials” section. Following the practice trials, the main task commenced. Throughout this task, checking responses, urge to check and distress ratings, answer accuracy, answer reaction time, corrugator supercilii activity, and skin conductance responses (not reported here) were recorded. At the end of the task, participants were presented with a condition-dependent message on the screen: the low-threat groups were informed they had gotten most of the trials correct, while the high-threat groups were informed that they got enough trials correct so would not receive the electric stimulation. Next, the avoidance task was then presented on the computer screen (not reported here).

Once the participants had finished the experiment, the research assistants removed the sensors from the participants, and a further series of questionnaires was completed (not reported here). Then, participants received a written debrief. Overall, the session took approximately 2 hrs in total.

### Data Acquisition and Reduction

All behavioural and self-report data were extracted using the E-Data Aid tool in E-Prime (Psychology Software Tools Ltd.).

#### Checking Behaviour

During the checking phase of each trial, participants’ choice to check or not and their reaction time were recorded. Checking behaviour represented the total frequency of checking responses per participant for each condition, defined by threat level (low vs. high), feedback type (feedback vs. no feedback), and shape type (identical vs. different), resulting in eight distinct conditions (low threat without feedback for identical stimuli; low threat with feedback for identical stimuli; low threat without feedback for different stimuli; low threat with feedback for different stimuli; high threat without feedback for identical stimuli; high threat with feedback for identical stimuli; high threat without feedback for different stimuli; high threat with feedback for different stimuli).

#### Ratings

Subjective ratings of the urge to check and distress were obtained using a low/high threat × feedback/no feedback factorial design, resulting in four unique conditions per participant (low threat without feedback; low threat with feedback; high threat without feedback; high threat with feedback). Threat was a between-participants factor and feedback was within-participants.

#### Accuracy

During the choice phase of each trial, participants’ accuracy while deciding whether the two shapes were identical or different, and their reaction time were recorded. Accurate responses during the choice phase were scored as binary (1 = accurate, 0 = inaccurate), and the proportion of accurate responses was calculated per participant for each of the eight conditions (as listed in the “Checking Behaviour” section above). For instance, the total number of accurate response trials was divided by the total number of trials per condition (i.e. five correct responses/six total trials = 0.83).

#### Corrugator Supercilii

The raw corrugator supercilii signal was root-mean-squared offline (Fridlund & Cacioppo, 1986). Corrugator supercilii activity was extracted for the feedback instruction cue period using R software (R Core Team, 2014) and averaged across the 2,000 ms window. These data were also baseline corrected by subtracting the 1,000 ms preceding each cue from a blank screen. Next, the data were *z*-scored to control for site effects due to the use of different electromyography (EMG) recording equipment and interindividual differences in corrugator supercilii activity that are unrelated to the task (i.e. muscle size; for similar analysis pipelines of the corrugator supercilii, see Morriss et al., 2020, 2022a).

For the feedback instruction cue period, corrugator supercilii trials were averaged per trial type for each participant, resulting in the eight conditions (as listed in the “Checking Behaviour” section).

### Missing Data and Exclusions

For the corrugator supercilii, five participants (one Reading, four Southampton) were missing all the data, and one participant (one Reading) was missing partial data due to recording errors. This leaves 202 participants with complete corrugator supercilii data. For the ratings of urge to check and distress, four participants (four Southampton) had partial data missinge, leaving 204 participants with complete rating data. There was no missing data for checking frequency, accuracy, or reaction times. No participants were excluded based on data quality, and no participants withdrew from the study.

### Data Analysis Plan

Prior to analyses, the data were assessed for normality and deemed suitable for parametric statistics. The task accuracy data were inspected for outliers.^1^ The descriptive stats were computed using SPSS 29.0.2.0 (SPSS, Inc., Chicago, IL, United States). MLM analyses were conducted using the *lmer* function in the lme4 package (Bates et al., 2015) in R version 4.4.1 (14 June 2024) within the mixed procedure. For the syntax, see the Supplemental Material.

We conducted separate MLMs for checking frequency, ratings of the urge to check, ratings of distress, answer accuracy during the choice phase, answer reaction time during the choice phase, and corrugator supercilii activity during the feedback instruction cue period.

In the analysis of each variable, threat level (two: low, high), feedback type (two: no feedback, feedback), shape type (two: identical, different), and site (two: Reading, Southampton) were entered at level 1 and individual participants at level 2 in the MLMs. Fixed effects included threat level, feedback type, shape type, and site. A diagonal covariance matrix was used for level 1 in all models. Random effects included participant, with a variance component covariance structure. We used a maximum likelihood estimator for the MLMs.

For the urge to check, accuracy during the choice phase, answer reaction time during the choice phase, and corrugator supercilii activity during the feedback instruction cue period, possible interactions included 2 threat level × 2 feedback type × 2 shape type × 2 site. For the ratings of urge to check and distress, the shape type factor was not included in the MLMs, as the ratings were collected at the end of each block of trials. Therefore, possible interactions for this variable included 2 threat level × 2 feedback type × 2 site.

Significant interactions were followed up using Tukey’s adjusted pairwise comparisons.

## Results

Results are organised by hypothesis (H1–H4) to capture how threat, feedback, threat × feedback interactions, and shape type influence the different measurements. If threat or feedback interacted with shape type, these results are presented within H1 to H3. Within each hypothesis, results are reported in the following order: checking frequency, self-report ratings (urge to check and distress), task performance (accuracy and reaction time), and physiological measures (corrugator supercilii). For descriptive statistics and violin plots of each measure, see the Supplemental Material. The effect sizes for all the MLMs are provided in Table 3.

**Table 3.** Marginal *R*^2^, Conditional *R*^2^, and Intraclass Correlation Coefficients with 95% Confidence Intervals from MLMs of Each Outcome Measure.

Models	Conditional *R*^2^	Marginal *R*^2^	ICC
Checking	.73 [0.69, 0.77]	.04 [0.02, 0.09]	.72 [0.67, 0.77]
Ratings: urge to check	.68 [0.62, 0.76]	.02 [0.01, 0.08]	.68 [0.59, 0.76]
Ratings: distress	.46 [0.36, 0.57]	.01 [0.01, 0.06]	.45 [0.32, 0.52]
Accuracy	.42 [0.38, 0.49]	.20 [0.17, 0.26]	.28 [0.18, 0.34]
Reaction times	.51 [0.45, 0.59]	.03 [0.02, 0.09]	.50 [0.43, 0.56]
Corrugator^a^	.06	NA	NA

*Note.* Conditional *R*^2^ reflects the variance explained by both fixed slopes (threat level, feedback, shape type, site) and random intercepts (individual subjects). Marginal *R*^2^ represents the variance explained by fixed slopes alone (threat level, feedback, shape type, site). The ICC indicates the variance explained by random intercepts (between-subject variability), independent of fixed effects. Estimates are presented with 95% confidence intervals. Models for checking, accuracy, reaction time and corrugator included fixed effects for threat level × feedback × shape type × site; models for urge to check and distress included fixed effects for threat level × feedback × site. ICC: intraclass correlation coefficient; MLM: multilevel model.

a Marginal *R*^2^ and ICCs could not be computed for the corrugator model as corrugator values were *z*-scored across all participants, removing between-subject variance.

### H1: Effects of Threat Level

#### Checking Frequency

There was no significant difference in checking frequency between the high-threat (*M* = 4.08, SE = 0.45) and low-threat (*M* = 3.09, SE = 0.44) conditions, *F*(1, 208) = 2.55, *p* = .112.

There was a significant interaction between site and threat level for checking frequency, *F*(1, 208) = 4.79, *p* = .030 (see Figure 2). At Reading, participants checked more frequently in the high-threat condition (*M* = 5.03, SE = 0.62) compared to the low-threat condition (*M* = 2.69, SE = 0.61), *p* = .040. However, at Southampton, there was no significant difference between high-threat (*M* = 3.13, SE = 0.64) and low-threat (*M* = 3.50, SE = 0.62) conditions, *p* = .977.

**Figure 2.**
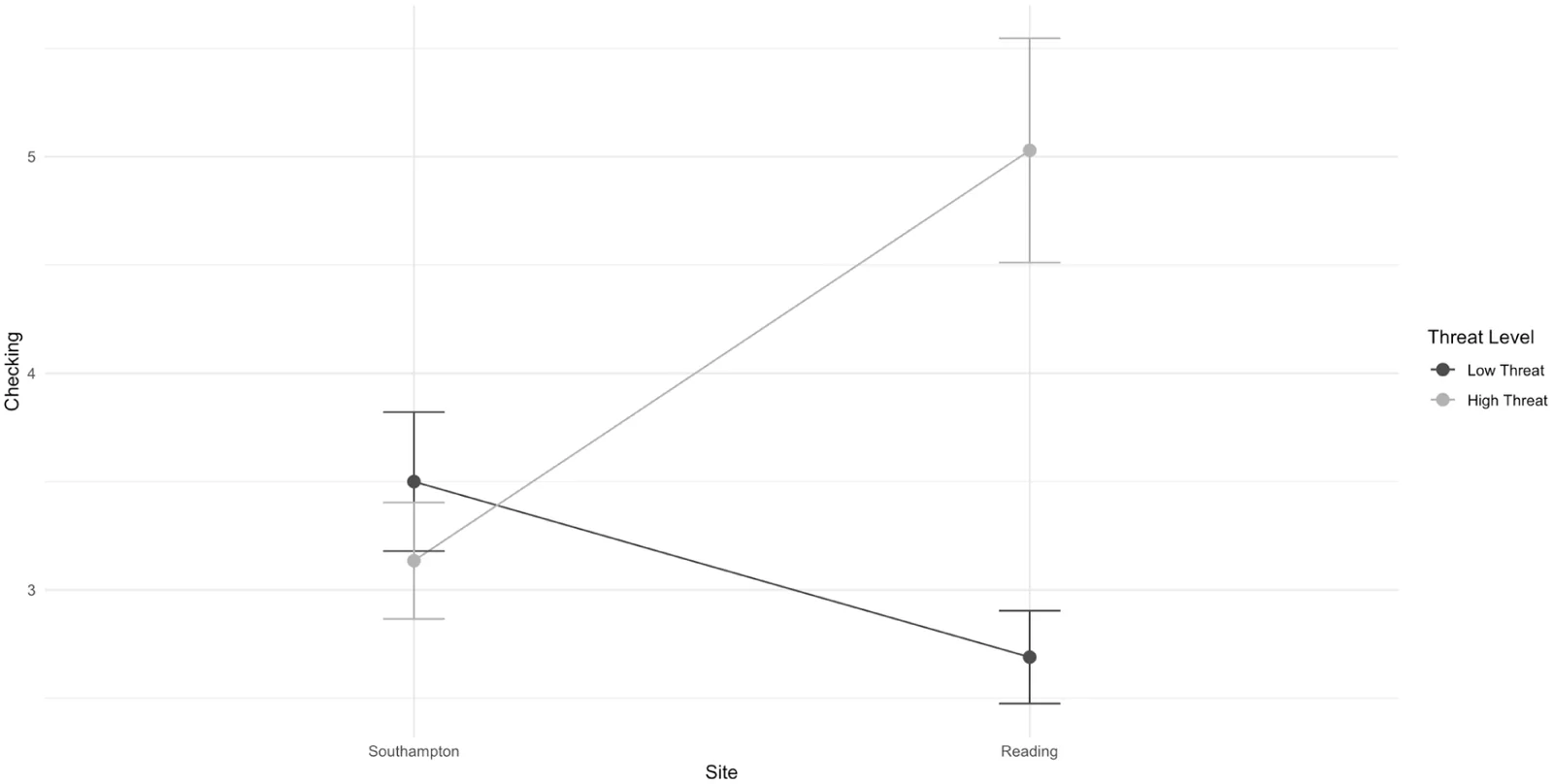
The interaction plot demonstrates the effect of threat level (low-threat or high-threat) and site (Reading or Southampton) on checking frequency. Participants checked more frequently in the high-threat condition compared to the low-threat condition in Reading. The error bars represent ±1 SEM. *Note.* SEM: standard error of the mean.

#### Ratings

##### Urge to Check

The high-threat (*M* = 30.55, SE = 2.28) and low-threat (*M* = 29.02, SE = 2.24) conditions did not have a significant effect on the participants’ urge to check, *F*(1, 207.51) = 0.24, *p* = .628.

##### Distress

The high-threat (*M* = 24.48, SE = 1.98) and low-threat (*M* = 22.29, SE = 1.95) conditions did not have a significant effect on the participants’ level of distress, *F*(1, 204.40) = 0.63, *p* = .427.

#### Task Performance Outcomes

##### Accuracy

There was no significant difference in task accuracy between high-threat (*M* = 0.76, SE = 0.01) and low-threat conditions (*M* = 0.75, SE = 0.01), *F*(1, 208) = 0.14, *p* = .707.

However, there was a significant interaction between the threat level and shape type, *F*(1, 624) = 9.03, *p* = .003 (see Figure 3). Follow-up tests revealed that both in the high-threat (*p* < .001) and low-threat conditions (*p* < .001), participants were more accurate in responding to identical shapes (*M* = 0.88, SE = 0.02; 0.84, SE = 0.02) when compared to different shapes (*M* = 0.63, SE = 0.02; 0.67, SE = 0.02). Participants were also more accurate in the high-threat condition (*M* = 0.88, SE = 0.01) compared to the low-threat condition (*M* = 0.84, SE = 0.01) when responding to the identical shapes, *p* = .010. However, there was no significant difference between the conditions when responding to different shapes, *p* = .270.

**Figure 3.**
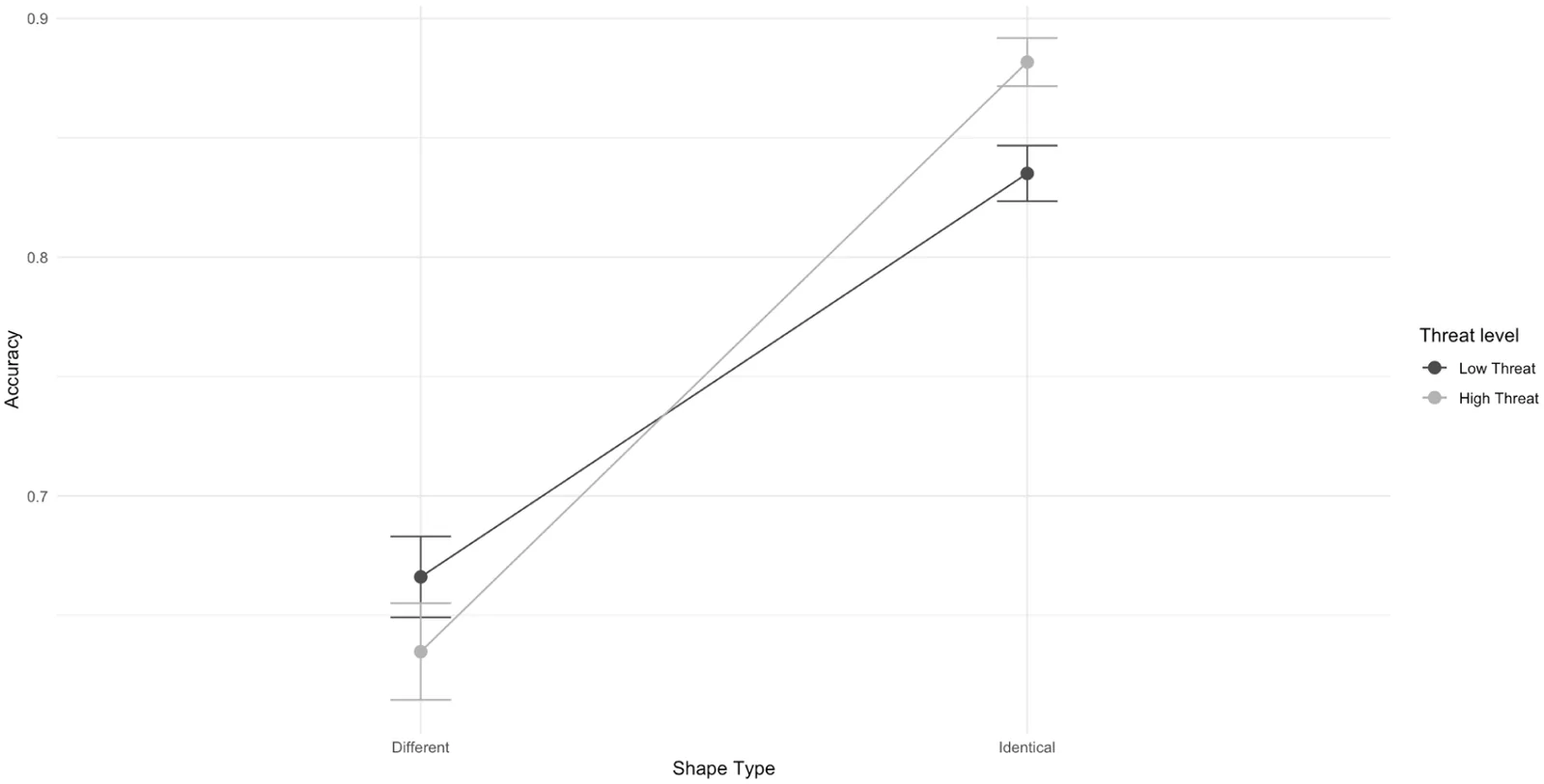
The interaction plot demonstrates the effect of threat level (low-threat or high-threat) and shape type (identical or different) on accuracy. Participants in the high-threat condition showed greater accuracy for identical shapes compared to those in the low-threat condition. The error bars represent ±1 SEM. *Note.* SEM: standard error of the mean.

##### Reaction Time

Participants did not demonstrate a significant difference in their reaction times (ms) between the high-threat (*M* = 887.10, SE = 31.79) and the low-threat conditions (*M* = 891.96, SE = 31.18), *F*(1, 208) = 0.01, *p* = .912.

#### Physiological Measure

##### Corrugator Supercilii

There was no difference in participants’ corrugator supercilii activity in response to cues signalling feedback availability between the high-threat (*M* = 0.00, SE = 0.01) and low-threat conditions (*M* = 0.00, SE = 0.01), *F*(1, 810) = 0.00, *p* = .997.

### H2: Effects of Performance Feedback

#### Checking Frequency

There was no significant difference in checking frequency between the feedback (*M* = 3.47, SE = 0.33) and no-feedback (*M* = 3.71, SE = 0.33) conditions, *F*(1, 624) = 1.71, *p* = .192.

#### Ratings

##### Urge to Check

The feedback condition had a significant effect on the participants’ urge to check, *F*(1, 202.16) = 4.91, *p* = .028. Participants reported significantly higher levels of urge to check when feedback was available (*M* = 31.34, SE = 1.75) compared to when feedback was not available (*M* = 28.23, SE = 1.74).

There was a significant interaction between feedback type and site, *F*(1, 202.16) = 6.41, *p* = .012. Participants in Southampton demonstrated significantly higher levels of the urge to check when feedback was available (*M* = 31.35, SE = 2.51) compared to when no feedback was available (*M* = 24.68, SE = 2.50), *p* = .008. At Reading, this effect was not observed, *p* > .050.

##### Distress

The no-feedback (*M* = 23.16, SE = 1.64) and feedback (*M* = 23.61, SE = 1.63) conditions had no significant effect on the participants’ level of distress, *F*(1, 202.12) = 0.07, *p* = .790.

#### Task Performance Outcomes

##### Accuracy

There was no significant difference in task accuracy between feedback (*M* = 0.75, SE = 0.01) and no-feedback conditions (*M* = 0.76, SE = 0.01), *F*(1, 624) = 1.00, *p* = .319.

There was also a significant interaction between feedback condition and shape type, *F*(1, 624) = 5.64, *p* = .018 (see Figure 4). Analyses revealed that in the no-feedback condition, participants were more accurate when responding to identical shapes (*M* = 0.88, SE = 0.02) compared to different shapes (*M* = 0.64, SE = 0.02), *p* < .001. Similarly, in the feedback condition, participants were more accurate when responding to identical shapes (*M* = 0.84, SE = 0.02) compared to different shapes (*M* = 0.66, SE = 0.02), *p* < .001. While participants were marginally more accurate in responding to identical shapes in the no-feedback condition (*M* = 0.88, SE = 0.02) compared to the feedback condition (*M* = 0.84, SE = 0.02), these differences were not statistically significant, *p* = .086.

**Figure 4.**
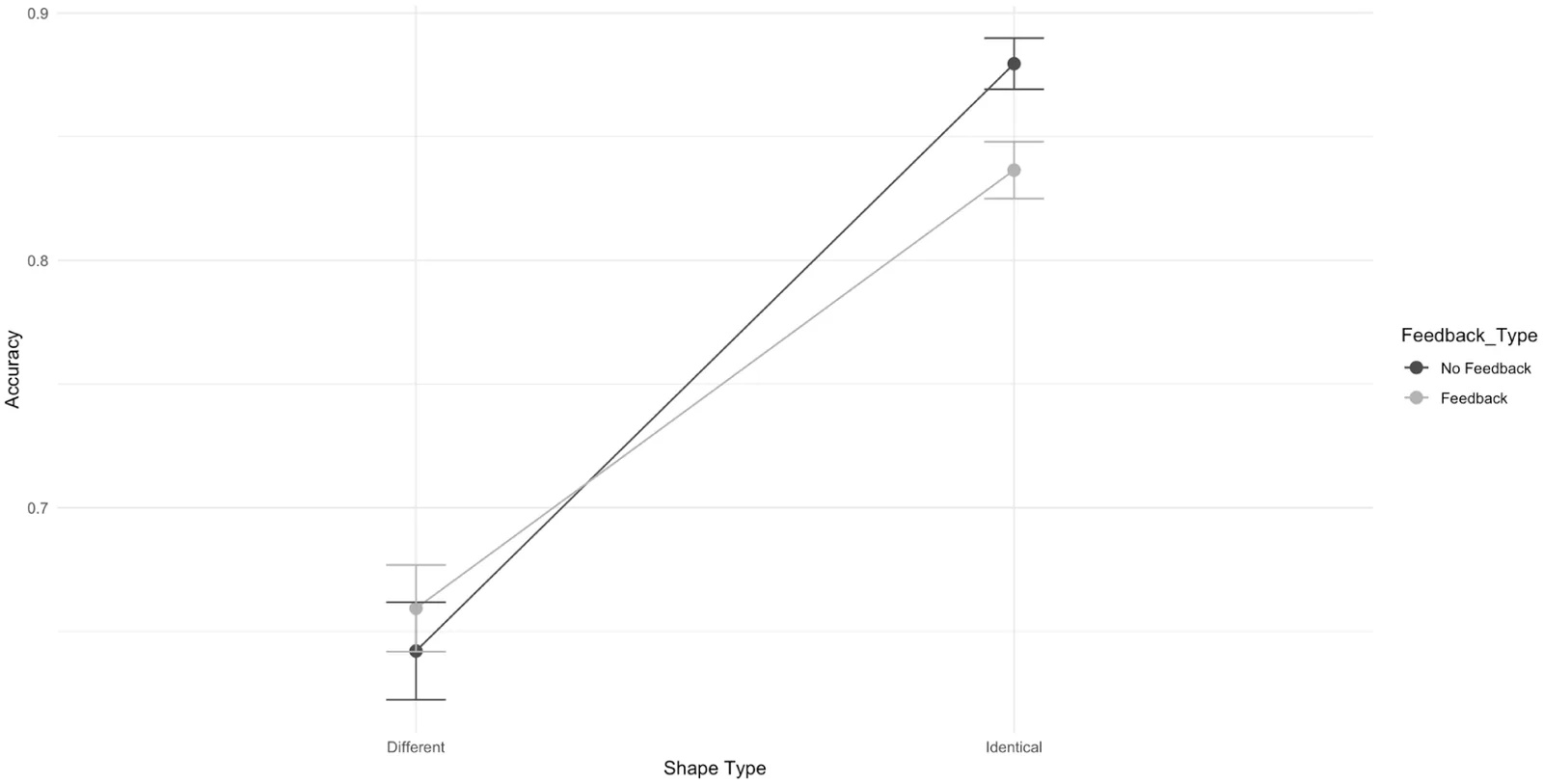
The interaction plot depicts the effect of feedback (no feedback vs. feedback) and shape type (identical vs. different) on accuracy. In the no-feedback versus feedback blocks, participants displayed greater accuracy for the identical shapes. The error bars represent ±1 SEM. *Note.* SEM: standard error of the mean.

##### Reaction Time

There was no significant difference in reaction times between feedback (*M* = 903.31, SE = 24.43) and no-feedback condition (*M* = 875.75, SE = 24.43), *F*(1, 624) = 1.92, *p* = .166.

However, there was a significant interaction between feedback condition and shape type for reaction time, *F*(1, 624) = 28.73, *p* < .001 (see Figure 5). When no feedback was available, participants responded significantly more quickly to different shapes (*M* = 825.30, SE = 28.26) compared to identical shapes (*M* = 926.20, SE = 28.26), *p* = .002. Conversely, when feedback was available, participants responded significantly more quickly to identical shapes (*M* = 847.15, SE = 28.26) compared to different shapes (*M* = 959.48, SE = 28.26), *p* < .001.

**Figure 5.**
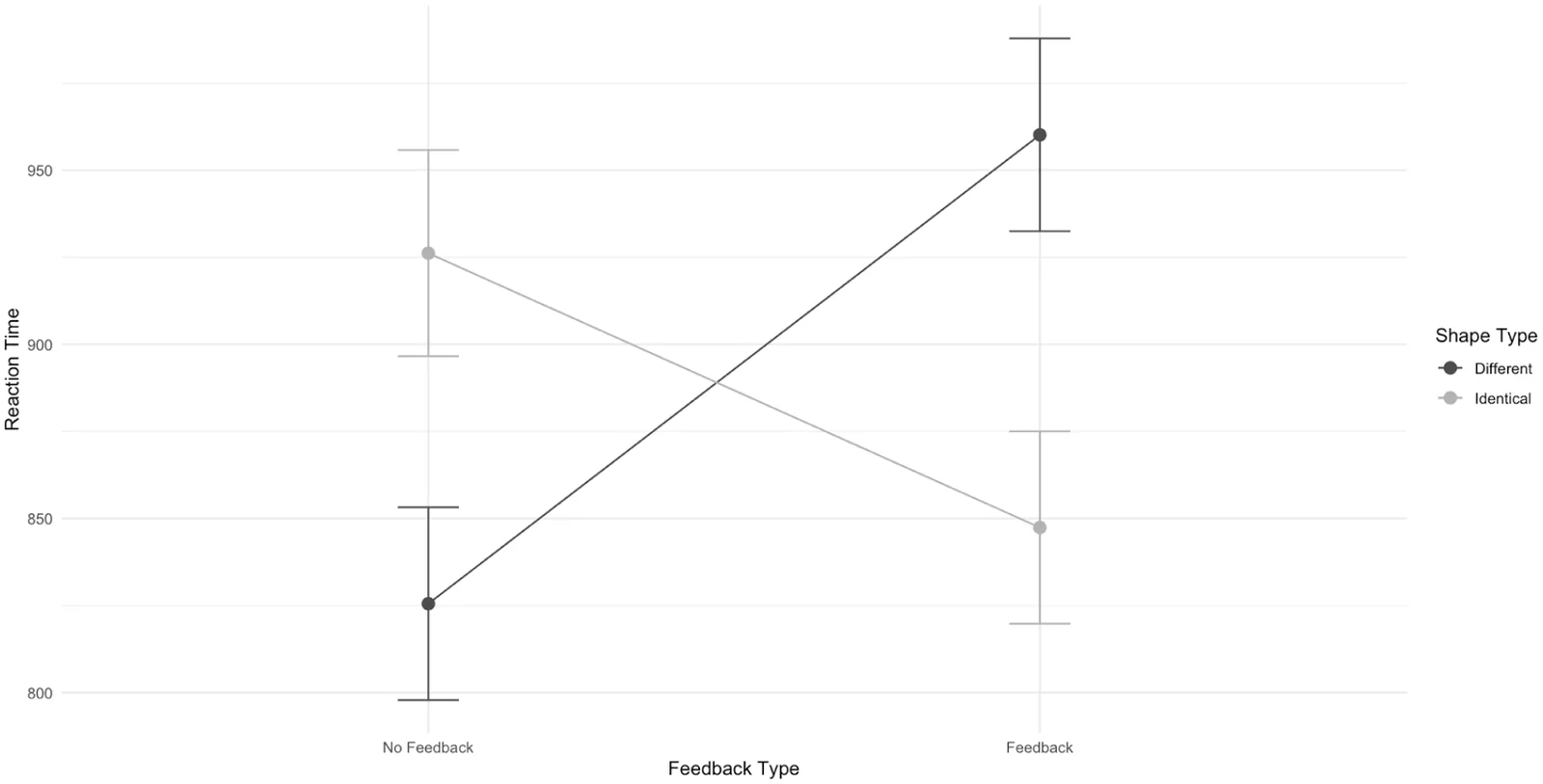
The interaction plot demonstrates the effect of shape type (identical or different) and feedback type (no feedback vs. feedback) on reaction time. Participants were quicker responding to different shapes when compared to identical shapes. Participants were quicker responding to identical shapes when feedback was available compared to when no-feedback was available. The error bars represent ±1 SEM. *Note.* SEM: standard error of the mean.

Participants were also significantly quicker in responding to identical shapes when feedback was available (*M* = 847.15, SE = 28.26) compared to when no feedback was available (*M* = 926.20, SE = 28.26), *p* = .028. Moreover, participants responded significantly more quickly to different shapes when no feedback was available (*M* = 825.30, SE = 28.26) compared to when feedback was available (*M* = 959.48, SE = 28.26), *p* < .001.

#### Physiological Measure

##### Corrugator Supercilii

Participants displayed significantly larger corrugator supercilii activity to the cue that signalled no feedback available (*M* = 0.02, SE = 0.01) compared to the cue that signalled feedback being available (*M* = −0.02, SE = 0.01), *F*(1, 810) = 4.30, *p* = .038 (see Figure 6A).

**Figure 6.**
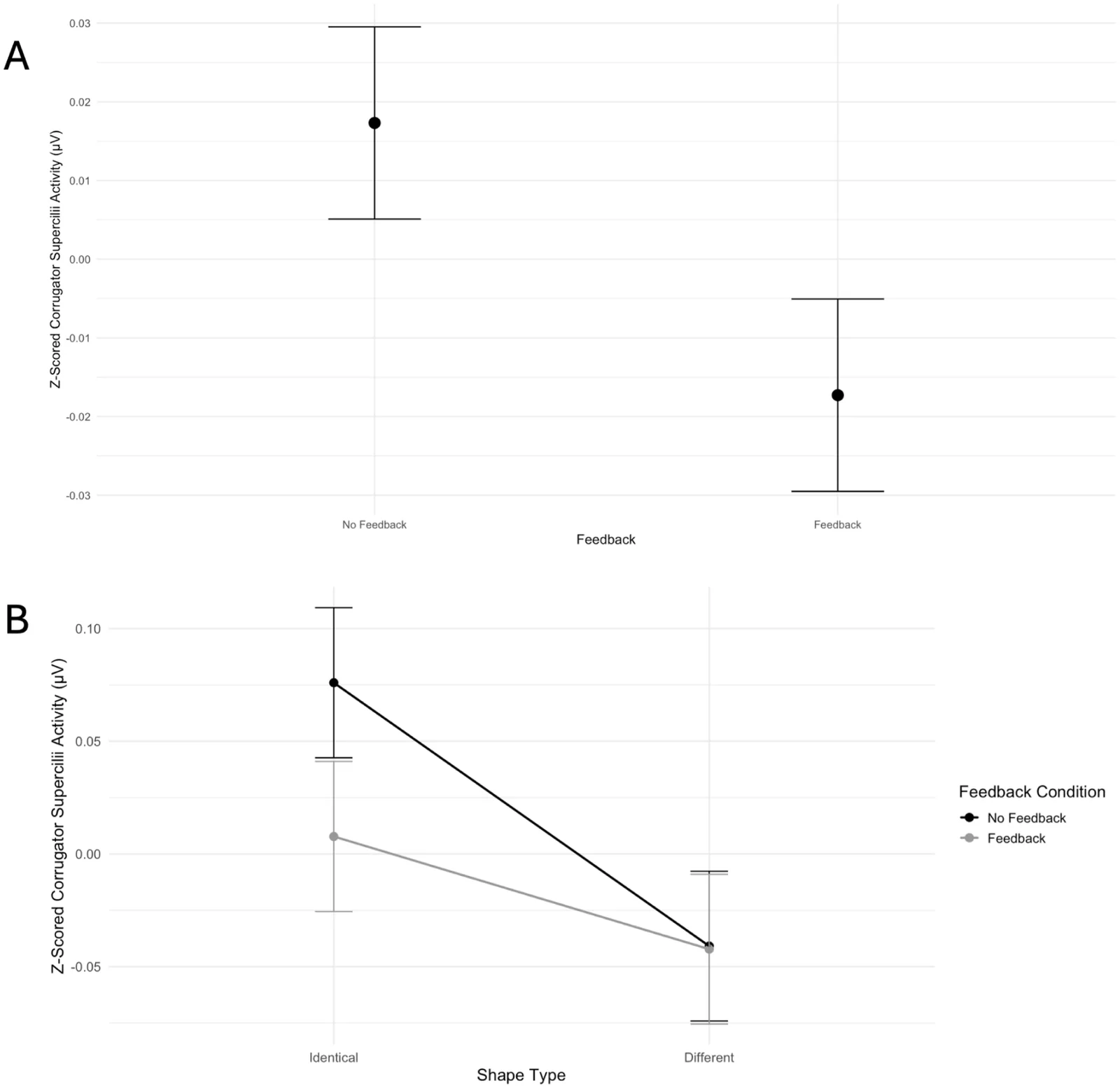
This plot demonstrates the difference in corrugator supercilii activity between no-feedback and feedback conditions (A). Participants had larger corrugator supercilii activity in response to the cue signalling no feedback compared to the cue signalling feedback was available. The interaction plot demonstrates the impact of feedback (no feedback vs. feedback) and shape type (identical vs. different) on corrugator supercilii activity (B). Participants displayed greater corrugator supercilii activity after viewing identical shapes in the no-feedback condition compared to the feedback condition. The error bars represent ±1 SEM. *Note.* SEM: standard error of the mean.

There was a significant interaction between the availability of feedback and the type of shape viewed, *F*(1,624) = 5.64, *p* = .018 (see Figure 6B). Further tests revealed that when participants viewed identical shapes, they exhibited significantly larger corrugator supercilii activity in response to the cue signalling no feedback (*M* = 0.08, SE = 0.02) compared to the cue signalling feedback (*M* = 0.01, SE = 0.02), *p* = .024. However, when the shapes were different, there was no significant difference in corrugator supercilii activity to the cue that signalled no feedback *(M* = 0.04, SE = 0.02) and feedback *(M* = 0.04, SE = 0.02), *p* = 1.000.

In the no-feedback condition, participants exhibited significantly larger corrugator supercilii activity in response to cues signalling feedback availability after viewing identical shapes (*M* = 0.08, SE = 0.02) compared to different shapes (*M* = −0.04, SE = 0.02), *p* < .001. However, there was no significant difference in corrugator supercilii activity after viewing identical shapes (*M* = 0.01, SE = 0.02) versus different shapes (*M* = −0.04, SE = 0.02) in the feedback condition, *p* = .159.

### H3: Interaction Between Threat Level and Performance Feedback

There were no significant interactions between threat level (high-threat or low-threat) and performance feedback (no-feedback or feedback) for any of the measures, max *F* = 3.90.

### H4: Exploratory Effects of Shape Type

#### Checking Frequency

There was a significant effect of shape type on checking frequency, *F*(1, 624) = 4.16, *p* = .042. Participants checked more frequently when the shapes were identical (*M* = 3.78, SE = 0.33) compared to when the shapes were different (*M* = 3.40, SE = 0.33).

#### Task Performance Outcomes

##### Accuracy

Participants were significantly more accurate in their responses when presented with identical shapes (*M* = 0.86, SE = 0.01) compared to different shapes (*M* = 0.65, SE = 0.01) during the task, *F*(1, 624) = 263.73, *p* < .001.

##### Reaction Time

There was no significant difference in the reaction time when making a decision following identical (*M* = 886.67, SE = 24.43) versus different shapes (*M* = 892.39, SE = 24.43), *F*(1, 624) = 0.08, *p* = .774.

#### Physiological Measure

##### Corrugator Supercilii^2^

Participants had a significantly larger corrugator supercilii activity in response to cues signalling feedback availability after viewing identical shapes (*M* = 0.04, SE = 0.01) compared to different shapes (*M* = −0.04, SE = 0.01), *F*(1, 810) = 24.72, *p* < .001.

### Post hoc Analyses

#### Controlling for OCI-R

To ensure that individual differences in OCI-R did not drive the observed effects, we conducted the MLMs again with OCI-R scores included as a covariate. All of the reported main effects and interactions above remained significant when controlling for OCI-R scores (*p*’s < .05). See Supplemental Table S3 for standardised betas, standard errors, and 95% confidence intervals for all main effects and significant interactions, and Supplemental Table S4 for the corresponding estimates when controlling for OCI-R scores.

## Discussion

The present study examined how different levels of threat (low: receiving a performance score; high: a potential electric shock based on task performance score) and performance feedback (no feedback, feedback) during a visual discrimination and checking task contribute to checking behaviour, subjective levels of the urge to check and distress, task accuracy and reaction time, and corrugator supercilii activity. Higher versus lower-threat level increased checking behaviour at one site but not the other. Furthermore, higher versus lower-threat level improved task performance for identifying identical shapes versus different shapes. In addition, no feedback, compared to feedback, was associated with greater corrugator supercilii activity and improved task performance when identifying identical shapes versus different shapes. Taken together, these findings provide insight into how factors such as threat level and performance feedback differently impact checking behaviour and its concomitants.

There was partial evidence for the hypothesis that higher-threat versus low-threat level would lead to greater checking frequency, in line with previous research (Li et al., 2016; Morein-Zamir et al., 2018). For instance, at the Reading site, participants in the high-threat condition, compared to those in low-threat condition, checked significantly more during the task. However, this effect was not observed for the Southampton site. It is not entirely clear why there were differences in checking frequency based on the threat level condition across sites, given that the participants across both sites reported similar pain ratings to the electric stimulation. There were differences in the equipment and electric stimulation location (fingers, wrist) for both sites, which may have introduced variation in the level of experienced threat. Considering prior research from Clair et al. (2013) and Wake et al. (2022), it is possible that the low-threat condition may still be threatening for participants who fear making mistakes, leading to increased checking behaviours, and as such, this may have possibly underscored the lack of significant differences in checking frequency between threat conditions. Furthermore, research from Naylor et al. (2015) found that individuals react differently to task demand, as individual traits can influence perception of whether the task is considered a challenge or a threat. Future research on the role of threat in checking behaviour across community samples may benefit from recording and controlling for such individual differences.

Additionally, there was little evidence of main effects of threat level impacting other measures during the task such as subjective ratings of distress and the urge to check, accuracy, reaction times, or corrugator supercilii activity. Although, there were interactions between threat level and shape type during the task for accuracy, such that participants in the high threat, compared to the low-threat group, were more accurate at identifying identical shapes. This finding is in line with previous research demonstrating how threat of shock can improve cognitive performance (for discussion see, Torissi et al., 2016). In sum, while there was some impact of threat level upon the measures, the number of null effects across measures suggest that the threat manipulation may have been relatively weak, at least across sites and participants.

Contrary to our hypothesis, there were no main effects of performance feedback type (e.g. no feedback, feedback) on actual checking behaviour, distress, accuracy, or reaction time during the task. It was hypothesised that the urge to check would be higher for feedback absent, compared to feedback present conditions, similar to findings from Parrish and Radomsky (2011). However, this was not observed in the current study. Instead, participants from one site reported higher levels of the urge to check when feedback was available, compared to when feedback was absent. Such findings may reflect individual differences in interpreting feedback. For example, findings from Anseel and Lievens (2007) found some individuals will actively seek feedback in order to improve or to have knowledge about their performance, while others may fear the outcome and consequently avoid feedback. Further research is required to tease apart these individual differences in sensitivity to feedback availability, as this may be useful for identifying different underlying biases that drive the urge to check in general and in relation to compulsions in OCD.

Analyses of shape type were performed to confirm that the task functioned as expected. Participants checked more frequently when the shapes were identical compared to when the shapes were different. Similar results have been observed in subclinical and clinical OCD populations (see Rotge et al., 2008), which has been interpreted to reflect higher feelings of doubt, even in contexts where visual discrimination is relatively easy or unambiguous. In this study, task accuracy was higher for identical compared to different shapes. This finding is in line with prior research, such as Wake et al. (2022), who observed the same pattern of results. In sum, these findings suggest that the visual discrimination and checking task was operating as expected.

Exploratory results of shape type also emerged for reaction time. Task reaction time was slower for identical shapes in no feedback blocks, compared to feedback blocks, whereas reaction time was faster for the different shapes in the no feedback blocks, compared to the feedback blocks. The effects of performance feedback and shape type on reaction time may be related to the higher rate of checking for the identical versus different shapes observed in this study. Although, post hoc correlations between checking frequency and reaction time did not support this interpretation (see Supplemental Table S5).

Interestingly, in this study for the corrugator supercilii, there was an interaction between performance feedback and shape type, where greater corrugator supercilii activity was observed for no feedback, compared to the feedback, after viewing identical shapes. Overall, these findings for the corrugator supercilii in this study match a previous study that found greater corrugator supercilii activity to cues signalling that checking was unavailable versus available (Wake et al., 2022). The convergent findings for the corrugator supercilii across these different types of task manipulation suggest that the corrugator supercilii may be an important measure for checking-related phenomena. Post hoc correlations between task ratings of distress and corrugator supercilii were not significant (see Supplemental Material). Thus, at this present time, it is unclear what the corrugator supercilii is tracking, whether it be distress, effort, or both, during checking tasks. This novel study has several strengths. The study was well powered and the visual discrimination and checking task passed manipulation checks. More specifically, the task operated as expected, with the identical shapes versus different shapes eliciting greater checking behaviours (Rotge et al., 2008) and higher accuracy (Wake et al., 2022). Furthermore, the study used a multimethod approach in a community sample to address an understudied area of research, namely the impact of threat level and the presence or absence of feedback on checking behaviour and its associated concomitants. There has been a lack of basic research on these factors and their role in checking behaviours within community samples. This makes it difficult to ascertain how checking behaviours operate across humans generally, and how these factors may contribute to the development of compulsive checking in clinical populations with OCD.

The study also had some limitations. Firstly, effects of threat on checking behaviour were only observed for one research site, suggesting that the high-threat condition did not reliably induce threat across the two research sites. The electric stimulation build-up procedure created comparable pain ratings across sites. However, the equipment used and location of the electric stimulation (i.e. finger, wrist) varied across the sites. Thus, future studies should adapt the visual discrimination and checking task to investigate checking behaviour with further optimised operationalisations of threat (e.g. variable levels of electric stimulation or more ecologically valid cost that are within-participant and presented throughout the task). Secondly, the visual discrimination and checking task, along with the threat manipulation of the electric stimulation may not have created an ecologically relevant task, as it likely does not directly capture the types of threat contexts associated with compulsive checking (e.g. responsibility of harm, uncertainty about safety or memory distrust; see Bucarelli & Purdon, 2016). Thirdly, the ratings of distress and the urge to check were only recorded at the end of each block of trials for the feedback and no feedback conditions. Collecting the distress and urge to check ratings at more time points throughout the task may improve the power of detecting effects of threat level and feedback. Lastly, the recruitment of a general community sample, rather than a screened non-clinical sample, may have introduced further noise into the data. For instance, the sample included a portion of participants who met criteria on the OCI-R questionnaire for clinically relevant OCD symptoms. Importantly, the results remained the same when controlling for the OCI-R questionnaire.

To conclude, the results here provide partial evidence that higher versus lower-threat level is associated with greater checking behaviour, as it was only observed at one site. In addition, higher versus lower-threat level is associated with improved task performance when identifying identical versus different shapes. Furthermore, the absence of feedback, compared to feedback is associated with greater corrugator supercilii activity and improved task performance when identifying identical versus different shapes. Overall, these findings provide insight into how factors such as threat level and performance feedback differently impact checking behaviour and its concomitants in community samples. Further research is warranted to assess whether these threat- and performance feedback-related effects are exacerbated in clinical samples with OCD.

## Supplemental Material

sj-docx-1-qjp-10.1177_17470218261456728 – Supplemental material for The Impact of Threat Level and Performance Feedback on Checking Behaviour and Its Concomitants: A Multi-Site Study Supplemental material, sj-docx-1-qjp-10.1177_17470218261456728 for The Impact of Threat Level and Performance Feedback on Checking Behaviour and Its Concomitants: A Multi-Site Study by Sruthi Sridhar, Bronwyn Steenekamp, Anthea Manuel, Belkis Sayin, Nicolò Biagi, Shannon Wake and Jayne Morriss in Quarterly Journal of Experimental Psychology

## Notes

1. For the task accuracy data, there were four participants who had average task accuracy ⩽45%. However, removing these participants did not change the results for task accuracy or reaction time. Therefore, these participants were retained for completeness.

2. There was a four-way interaction between check allocation, feedback, shape, and site that was statistically significant for corrugator activity, but this was not explored further due to its complexity and limited interpretability within the scope of this study.
