# Supplementary material for: The Impact of Threat Level and Performance Feedback on Checking Behaviour and Its Concomitants: A Multi-Site Study
Source: Q J Exp Psychol (Hove). 2026 May 26;79(11):3034–49. doi: 10.1177/17470218261456728 (PMC13675593; doi:10.1177/17470218261456728)
Supplement: Supplementary file 1 [file sj-docx-1-qjp-10.1177_17470218261456728.docx]

Supplementary Material for:

**The impact of threat level and performance feedback on checking behaviour and its concomitants: A multi-site study**

Sruthi Sridhar ^1^*, Bronwyn Steenekamp ^2^*, Anthea Manuel ^2^, Belkis Sayin ^2^, Nicolo Biagi ^3^, Shannon Wake^1^, & Jayne Morriss^2^**

^1^ School of Psychology and Clinical Language Sciences, University of Reading, Reading, UK

^2^ School of Psychology, Faculty of Environmental and Life Sciences, University of Southampton, Southampton, UK

^3^ Henley Business School, Business Informatics Systems and Accounting, Informatics Research Centre, University of Reading, UK

*Joint first authorship

** Correspondence:

Jayne Morriss

School of Psychology

B44 University Rd

University of Southampton

Southampton

SO17 1PS

[j.morriss@soton.ac.uk](mailto:j.morriss@soton.ac.uk)

Supplementary Table 1. Descriptive statistics for checking frequency, accuracy, reaction time, and corrugator supercilii activity.

|  | Reading | | | | | | | | Southampton | | | | | | | |
| --- | --- | --- | --- | --- | --- | --- | --- | --- | --- | --- | --- | --- | --- | --- | --- | --- |
|  | Low threat | | | | High threat | | | | Low threat | | | | High threat | | | |
|  | No feedback | | Feedback | | No feedback | | Feedback | | No feedback | | Feedback | | No feedback | | Feedback | |
|  | Identical | Diff | Identical | Diff | Identical | Diff | Identical | Diff | Identical | Diff | Identical | Diff | Identical | Diff | Identical | Diff |
| Checking Frequency | 2.70 (2.94) | 2.69 (3.03) | 3.17 (3.84) | 2.20 (2.69) | 5.58 (10.49) | 5.08 (6.75) | 5.00 (6.80) | 4.46 (4.87) | 3.71 (4.94) | 3.71 (5.63) | 3.38 (4.01) | 3.19  (3.80) | 2.96 (3.76) | 3.24 (3.63) | 3.70 (4.45) | 2.64 (3.30) |
| Accuracy | 0.86 (0.14) | 0.71 (0.23) | 0.81 (0.15) | 0.70 (0.21) | 0.92 (0.12) | 0.63 (0.28) | 0.89 (0.13) | 0.67 (0.29) | 0.85 (0.18) | 0.61 (0.30) | 0.82 (0.19) | 0.64 (0.23) | 0.89 (0.13) | 0.61 (0.32) | 0.82 (0.17) | 0.63 (0.27) |
| Reaction time | 916.39 (546.66) | 787.90 (350.04) | 833.12 (368.66) | 987.37 (564.81) | 920.91 (341.07) | 918.31 (526.26) | 901.14 (403.18) | 954.54 (270.82) | 950.78 (367.86) | 831.30 (311.57) | 847.77 (382.84) | 981.02 (354.22) | 916.72 (428.82) | 763.68 (370.30) | 806.54 (443.47) | 914.98 (343.56) |
| Z-scored corrugator supercilii activity (μV) | 0.02  (0.21) | -0.05 (0.21) | 0.09 (0.24) | -0.07  (0.23) | 0.08 (0.25) | 0.002 (0.19) | -0.07  (0.25) | -0.02  (0.25) | 0.11 (0.26) | -0.07  (0.22) | -0.02  (0.26) | -0.02 (0.24) | 0.09 (0.30) | -0.04 (0.26) | 0.03 (0.25) | -0.07 (0.22) |

Supplementary Figures. Violin plots of all measures: checking frequency, ratings of the urge to check and distress, accuracy, reaction time, and corrugator supercilii activity.

*Note, the solid black line within each violin plot represents the mean value for that condition. F = feedback, NF = no feedback.*

*Note, the solid black line within each violin plot represents the mean value for that condition. F = feedback, NF = no feedback.*

*Note, the solid black line within each violin plot represents the mean value for that condition. F = feedback, NF = no feedback.*

*Note, the solid black line within each violin plot represents the mean value for that condition. F = feedback, NF = no feedback.*

*Note, the solid black line within each violin plot represents the mean value for that condition. F = feedback, NF = no feedback.*

**Questionnaires**

***Generalized Anxiety Disorder 7-Item Scale***

We administered the Generalized Anxiety Disorder 7-Item Scale to assess generalized anxiety disorder symptoms (GAD-7; Spitzer et al., 2006). The items are rated on a 4-point Likert Scale (0-3). Cutoff points of 5, 10, and 15 represent mild, moderate, and severe levels of anxiety, respectively. See Supplementary Table 2 for the mean, standard deviation and site comparisons for each questionnaire. The internal reliability of GAD-7 for each site: Reading: α =.89; Southampton: α = .87.

***Intolerance of Uncertainty Scale***

We administered the short version of the Intolerance of Uncertainty Scale (IUS-12; Carleton et al., 2007). The 12 items are rated on a 5-point Likert Scale. The internal reliability of IUS-12 for each site: Reading: α = .88; Southampton: α = .91.

***Obsessive Compulsive Inventory – Revised***

We administered the Obsessive Compulsive Inventory – Revised to assess obsessive and compulsive symptoms (OCI-R; Foa et al., 2002). The questionnaire contains 18 items rated on a 5-point Likert scale (0-4) (Foa et al., 2002). A cutoff score of 21 is used to determine clinical levels of OCD. The internal reliability of OCI-R for each site: Reading: α =.87; Southampton: α = .88.

***Panic Disorder Severity Scale***

We administered the Panic Disorder Severity Scale to assess panic symptoms (PDSS-SR; Shear et al., 1997). The PDSS-SR has 7 items rated on a 5-point Likert Scale (0-4). A Cutoff score of 8 is used to determine clinical levels of panic disorder (Shear et al., 2001). The internal reliability of PDSS-SR for each site: Reading: α =.87; Southampton: α = .91.

***Patient Health Questionnaire-9***

We administered the Patient Health Questionnaire-9 to assess depression symptoms (PHQ-9; Kroenke et al., 2001). The PHQ-9 has 9 items rated on 4-point Likert Scale (0-3). Scores between 0-3 indicate minimal depression, 5–9 indicate mild depression, 10-14 indicate moderate depression, 15-19 indicate moderately severe depression, and 20-27 indicate severe depression (Kroenke et al., 2001). The internal reliability of PHQ-9 for each site: Reading: α =.87; Southampton: α = .87.

***Posttraumatic Stress Disorder Checklist***

We administered the Posttraumatic Stress Disorder Checklist to assess posttraumatic stress symptoms (PCL-5; Weathers et al., 2013). The PCL-5 has 20 items rated on a 5-point Likert Scale (0-4). A cutoff score of 31 is recommended to determine clinical levels of PTSD in community populations (Ashbaugh et al., 2016; Forkus et al., 2023). The internal reliability of PCL-5 for each site: Reading: α =.93; Southampton: α = .95.

***Social Phobia Inventory***

We administered the Social Phobia Inventory to assess social phobia symptoms (SPIN; Connor et al., 2000). The questionnaire contains 17 items rated on a 5-point Likert scale (0-4) (Connor et al., 2000). A cutoff score of 19 to determine clinical levels of social phobia (Connor et al., 2018). The internal reliability of SPIN for each site: Reading: α =.89; Southampton: α = .90.

***Trait Anxiety Inventory***

We administered the short version of the State and Trait (trait only recorded) Anxiety Inventory to assess trait anxiety (STAIT-5; Zsido et al., 2020). The 5 items are rated on a 4-point Likert Scale. The internal reliability of STAIT-5 for each site: Reading: α =.79; Southampton: α = .87.

Supplementary Table 2. Means, standard deviations and site comparisons of questionnaires.

|  |  |  | Site comparisons | | |  |
| --- | --- | --- | --- | --- | --- | --- |
|  | Reading | Southampton | t | df | p | d |
| GAD-7 (mean (SD)) | 8.46 (5.56) | 8.30 (4.90) | 0.23 | 205 | .821 | 0.03 |
| IUS-12 (mean (SD)) | 30.43 (9.15) | 28.41 (9.30) | 1.58 | 205 | .115 | 0.22 |
| OCI-R (mean (SD)) | 25.40 (12.37) | 22.60 (11.94) | 1.66 | 206 | .099 | 0.23 |
| PCL-5 (mean (SD)) | 27.61 (17.66) | 25.87 (18.76) | 0.69 | 204 | .493 | 0.10 |
| PDSS-SR (mean (SD)) | 4.46 (4.43) | 4.45 (4.71) | 0.03 | 205 | .979 | 0.00 |
| PHQ-9 (mean (SD)) | 9.57 (6.39) | 8.67 (5.94) | 1.04 | 204 | .299 | 0.15 |
| SPIN (mean (SD)) | 22.33 (12.31) | 22.12 (11.97) | 0.13 | 206 | .900 | 0.02 |
| STAIT-5 (mean (SD)) | 11.80 (3.61) | 11.30 (3.81) | 0.98 | 205 | .329 | 0.14 |

Supplementary Table 3. Standardised Betas from Main Effects and Interaction from Multilevel Models for Each Measure

|  | Standardised b (β) | Standardised SE | Standardised CI |
| --- | --- | --- | --- |
| **Checking Frequency** |  |  |  |
| Threat | -0.09 | 0.19 | [-0.47 – 0.29] |
| Feedback | -0.10 | 0.10 | [-0.30 – 0.10] |
| Shape | 0.00 | 0.10 | [-0.20 – 0.20] |
| Site x Threat | 0.56 | 0.27 | [0.03 – 1.10] |
| **Ratings: Urge to Check** |  |  |  |
| Threat | -0.09 | 0.20 | [-0.48 – 0.30] |
| Feedback | 0.27 | 0.11 | [0.04 – 0.49] |
| Site x Feedback | -0.24 | 0.16 | [-0.56 – 0.07] |
| **Ratings: Distress** |  |  |  |
| Threat | -0.08 | 0.20 | [-0.48 – 0.31] |
| Feedback | -0.06 | 0.15 | [-0.35 – 0.23] |
| **Accuracy** |  |  |  |
| Threat | -0.04 | 0.18 | [-0.38 – 0.31] |
| Feedback | 0.10 | 0.15 | [-0.19 – 0.39] |
| Shape | 0.96 | 0.15 | [0.67 – 1.26] |
| Site | 0.41 | 0.17 | [0.07 – 0.75] |
| Shape x Threat | 0.22 | 0.21 | [-0.20 – 0.63] |
| Feedback x Shape | -0.23 | 0.21 | [-0.64 – 0.19] |
| **Reaction Times** |  |  |  |
| Threat | -0.17 | 0.20 | [-0.55 – 0.22] |
| Feedback | 0.37 | 0.14 | [0.10 – 0.64] |
| Shape | 0.29 | 0.14 | [0.02 – 0.56] |
| Feedback x Shape | -0.62 | 0.19 | [-1.00 – -0.24] |
| **Corrugator** |  |  |  |
| Threat | -0.08 | 0.20 | [-0.46 – 0.31] |
| Feedback | -0.51 | 0.20 | [-0.89 – -0.12] |
| Shape | -0.73 | 0.20 | [-1.12 – -0.34] |
| Feedback x Shape | 0.73 | 0.28 | [0.18 – 1.27] |

**Note.** β = standardised coefficient; SE = standardised standard error; CI = standardised 95% confidence interval.

Supplementary Table 4. Standardised Betas from Main Effects and Interaction from Multilevel Models for Each Measure while Controlling for OCI-R

|  | Standardised b (β) | Standardised SE | Standardised CI |
| --- | --- | --- | --- |
| **Checking Frequency** |  |  |  |
| Threat | -0.09 | 0.19 | [-0.47 – 0.29] |
| Feedback | -0.10 | 0.10 | [-0.30 – 0.10] |
| Shape | 0.00 | 0.10 | [-0.20 – 0.20] |
| Site x Threat | 0.56 | 0.27 | [0.02 – 1.09] |
| **Ratings: Urge to Check** |  |  |  |
| Threat | -0.09 | 0.20 | [-0.47 – 0.30] |
| Feedback | 0.27 | 0.11 | [0.04 – 0.49] |
| Site x Feedback | -0.24 | 0.16 | [-0.55 – 0.07] |
| **Ratings: Distress** |  |  |  |
| Threat | -0.07 | 0.19 | [-0.46 – 0.31] |
| Feedback | -0.07 | 0.15 | [-0.36 – 0.22] |
| **Accuracy** |  |  |  |
| Threat | -0.04 | 0.18 | [-0.38 – 0.31] |
| Feedback | 0.10 | 0.15 | [-0.19 – 0.39] |
| Shape | 0.96 | 0.15 | [0.67 – 1.26] |
| Site | 0.41 | 0.17 | [0.07 – 0.75] |
| Shape x Threat | 0.22 | 0.21 | [-0.20 – 0.63] |
| Feedback x Shape | -0.23 | 0.21 | [-0.64 – 0.19] |
| **Reaction Times** |  |  |  |
| Threat | -0.17 | 0.20 | [-0.55 – 0.21] |
| Feedback | 0.37 | 0.14 | [0.10 – 0.64] |
| Shape | 0.29 | 0.14 | [0.02 – 0.56] |
| Feedback x Shape | -0.62 | 0.19 | [-1.00 – -0.24] |
| **Corrugator** |  |  |  |
| Threat | -0.08 | 0.20 | [-0.46 – 0.31] |
| Feedback | -0.51 | 0.20 | [-0.46 – 0.31] |
| Shape | -0.73 | 0.20 | [-1.12 – -0.34] |
| Feedback x Shape | 0.73 | 0.28 | [0.18 – 1.27] |

**Note.** β = standardised coefficient; SE = standardised standard error; CI = standardised 95% confidence interval.

**Correspondence between measures**

*Correspondence between checking frequency and task performance outcomes:* To examine whether checking frequence was associated with task performance, parametric correlations were conducted between checking frequency, accuracy, and reaction time separately for each combination of feedback type and shape type, for the low and high threat groups (see Supplementary Table 5). Checking frequency was not significantly associated with accuracy in either threat condition. In the high threat condition, checking frequency was positively correlated with reaction time across all conditions, suggesting that participants who checked more frequently were slower to respond, but this did not translate into improved accuracy. Significant negative correlations were found between accuracy and reaction time in the low threat condition, under both feedback conditions for identical shapes suggesting that participants who responded more quickly were more accurate in these conditions. Similarly, a significant negative correlation was found between accuracy and reaction time in the high threat condition, but only during the feedback condition for different shapes.

**Supplementary Table 5. Correlations Between Checking Frequency and Task Performance by Condition**

| **Threat Condition** | **Feedback Condition** | **Shape Type** | **Checking x Accuracy** | **Checking x RT** | **Accuracy x RT** |
| --- | --- | --- | --- | --- | --- |
| **Low Threat** | No Feedback | Different | *r* = -0.02, *p* = 0.874 | *r* = 0.16, *p* = 0.102 | *r* = -0.04, p = 0.673 |
|  | No Feedback | Identical | *r* = 0.03, *p* = 0.750 | *r* = 0.08, *p* = 0.423 | *r* = -0.24*, p = 0.015 |
|  | Feedback | Different | *r* = -0.02, *p* = 0.827 | *r* = 0.07, *p* = 0.499 | *r* = -0.11, p = 0.260 |
|  | Feedback | Identical | *r* = 0.07, *p* = 0.480 | *r* = 0.04, *p* = 0.705 | *r* = -0.36***, p < .001 |
| **High Threat** | No Feedback | Different | *r* = 0.03, *p* = 0.733 | *r* = 0.23*, *p* = 0.022 | *r* = -0.09, p = 0.347 |
|  | No Feedback | Identical | *r* = -0.01, *p* = 0.884 | *r* = 0.34***, *p* < 0.001 | *r* = -0.09, p = 0.387 |
|  | Feedback | Different | *r* = -0.12, *p* = 0.229 | *r* = 0.27**, *p* = 0.006 | *r* = -0.25*, p = 0.012 |
|  | Feedback | Identical | *r* = 0.00, *p* = 0.966 | *r* = 0.33***, *p* = 0.001 | *r* = 0.06, p = 0.522 |

Note. Parametric correlations between checking frequency and task performance (accuracy and reaction time) across feedback and shape conditions, separated by threat group. *p < .05. **p < .01. ***p < .001.

*Correspondence between distress ratings and corrugator supercilii activity*: To examine whether corrugator supercilii activity was associated with self-reported distress, parametric correlations were conducted between corrugator supercilii activity and distress ratings separately for the feedback and no feedback conditions. Corrugator supercilii activity was not significantly correlated with distress ratings in either the no feedback condition (*r* = -.05, *p* = .332) or the feedback condition (*r* = -.01, *p* = .866).

**MLM lmer syntax**

*# Model 1 for Urge*
MLM_mod_1a_urge <- lmer(Urge ~ Threat * Feedback * Site + (1 | ParticipantID),
 data = df_ratings,
 REML = FALSE)

*# Model 1 for Distress*
MLM_mod_1b_distress <- lmer(Distress ~ Threat * Feedback * Site + (1 | ParticipantID),
 data = df_ratings,
 REML = FALSE)

*# Model 1 for Checking*
MLM_mod_1d_checking <- lmer(Checking ~ Threat * Feedback * Shape * Site + (1 | ParticipantID),
 data = df_checking,
 REML = FALSE)

*# Model 1 for Accuracy*
MLM_mod_1c_accuracy <- lmer(Accuracy ~ Threat Feedback * Shape * Site + (1 | ParticipantID),
 data = df_accuracy,
 REML = FALSE)

*# Model 1 for reaction times*
MLM_mod_1g_rt <- lmer(Reaction_Time ~ Threatn* Feedback * Shape * Site + (1 | ParticipantID),
 data = df_rt,
 REML = FALSE)

*# Model 1 for Corrugator*
MLM_mod_1e_corrugator <- lmer(Corr ~ Threat * Feedback * Shape * Site + (1 | ParticipantID),
 data = df_corrugator,
 REML = FALSE)
